# Diagnostic work-up of lipomatous tumors: a decision-making analysis among European sarcoma centers

**DOI:** 10.1186/s13244-025-02012-7

**Published:** 2025-06-14

**Authors:** Ali Naimi, Paul Martin Putora, Christian Rothermundt, Antonia Digklia, Jose Manuel Asencio, Sylvie Bonvalot, Florian Bösch, Anant Desai, Amer James Durrani, Haim Gutman, Daphne Hompes, Jens Jakob, Wolfram Trudo Knoefel, Elisabetta Pennacchioli, Piotr Rutkowski, Winan J. van Houdt, Barbara L. van Leeuwen, Stephan Waelti, Tim Steffen Fischer, Stefan Markart, Simon Wildermuth, Tobias Johannes Dietrich

**Affiliations:** 1 Radiology and Nuclear Medicine, Kantonsspital St. Gallen, St. Gallen, Switzerland; 2 Faculty of Medicine, University of Zurich, Zurich, Switzerland; 3 Department of Radiation Oncology, Kantonsspital St. Gallen, St. Gallen, Switzerland; 4 Division of Medical Oncology and Hematology, Kantonsspital St. Gallen, St. Gallen, Switzerland; 5 Sarcoma Center, Centre Hospitalier Universitaire Vaudois, University of Lausanne, Lausanne, Switzerland; 6 General Surgery III Department and Liver Transplant Unit, Hospital General Universitario Gregorio Marañón, Madrid, Spain; 7 Department of Surgical Oncology, Institut Curie, Paris, France; 8 Department of General, Visceral and Pediatric Surgery, University Medical Center Göttingen, Göttingen, Germany; 9 Midlands Abdominal and Retroperitoneal Sarcoma Unit (MARSU), University Hospitals Birmingham NHS Foundation Trust, Birmingham, United Kingdom; 10 Department of Plastic Surgery, University of Cambridge and Addenbrooke’s Hospital, Cambridge, UK; 11 Surgical Oncology Unit, Department of Surgery, Herzliya Medical Center, Herzliya, Israel; 12 Department of Surgical Oncology, University Hospitals Gasthuisberg, Leuven, Belgium; 13 Sarcoma Unit, Department of Surgery, University Medical Center and Medical Faculty Mannheim, University of Heidelberg, Mannheim, Germany; 14 Department of General, Visceral and Pediatric Surgery, University Hospital, Medical Faculty, Heinrich-Heine-University Duesseldorf, Duesseldorf, Germany; 15 Division of Melanoma, Sarcomas and Rare Tumors, IEO, European Institute of Oncology, IRCCS, Milan, Italy; 16 Department of Soft Tissue/Bone Sarcoma and Melanoma, Maria Sklodowska-Curie National Research Institute of Oncology, Warsaw, Poland; 17 The Division of Surgical Oncology, Netherlands Cancer Institute, Department of Medical Oncology, Amsterdam University Medical Center, Amsterdam, The Netherlands; 18 Department of Surgery, University Medical Center Groningen, Groningen, The Netherlands; 19 Radiology and Nuclear Medicine, Kantonsspital Winterthur, Winterthur, Switzerland

**Keywords:** Adipocytic soft-tissue tumor, Lipoma, Liposarcoma, Diagnostic management, Decision-making

## Abstract

**Objectives:**

Lipomatous soft-tissue tumors present a diagnostic burden. The aim of this work was to compare standard operating procedures (SOPs) for the diagnostic management of lipomatous soft-tissue tumors among European academic centers.

**Methods:**

Experts of the Soft Tissue and Bone Sarcoma Group of the European Organization for Research and Treatment of Cancer were asked for their SOPs in the diagnosis of adipocytic soft-tissue tumors in an otherwise healthy patient. The answers were converted to decision trees and subsequently compared using the objective consensus methodology. Mediastinal and retroperitoneal lipomatous tumors were excluded from the analysis.

**Results:**

The highest consensus (93%) among fourteen institutions was noted for evaluation with core needle biopsy (CNB) as SOP for lipomatous tumors located deep in tissue exceeding 7 cm and tumor-associated symptoms. Evaluation of heterogeneous features on imaging by CNB usually showed a consensus rate of at least 75%. Consensus was less likely for lipomatous tumors without symptoms or heterogeneous features. In these settings, CNB and follow-up were almost equally recommended. For lipomatous tumors smaller than 3 cm, without growth or symptoms, no localization in the trunk, and homogeneous imaging features, a consensus rate of 71% was achieved for follow-up.

**Conclusions:**

SOPs for diagnostic work-up of lipomatous tumors varied despite their geographical proximity. The highest consensus for biopsy was for deep large tumors with associated symptoms. For follow-up, consensus was shown for small homogenous tumors outside the trunk, without growth or symptoms. Consensus on resection involved homogeneous deeply located small tumors outside the trunk with growth and symptoms.

**Critical relevance statement:**

This study identifies the decision-making criteria with the highest consensus rate among participating academic sarcoma centers in diagnosing lipomatous tumors: tumors located deep in the tissue, a tumor size exceeding 7 cm, and associated symptoms emerge as pivotal criteria.

**Key Points:**

- Standard operating procedures for diagnostic work-up of lipomatous tumors among fourteen sarcoma centers were analyzed.
- Identified diagnostic criteria are: imaging features, size, growth, symptoms, superficial and trunk location.
- The highest consensus concerned recommending biopsies for deep tumors > 7 cm with associated symptoms.

**Graphical Abstract:**

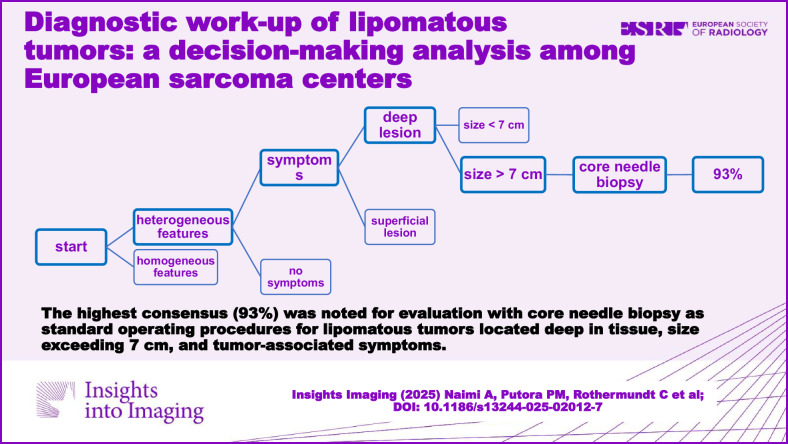

## Introduction

Lipomatous tumors mainly include lipomas, lipoma variants, atypical lipomatous tumors/well-differentiated liposarcomas and less frequently dedifferentiated liposarcomas [1]. Moreover, macroscopic fat-containing tumors detected on imaging, such as hibernomas, intramuscular hemangiomas, solitary fibrous tumors, myelolipomas, extramedullary hematopoiesis, and germ cell tumors, particularly teratomas and dermoid cysts, may mimic lipomas, atypical lipomatous tumors, and well-differentiated liposarcomas.

Lipomatous tumors encompass a spectrum of adipocytic soft-tissue tumors, posing significant diagnostic challenges due to their diverse histological subtypes and overlapping clinical and radiological features [2, 3]. Lipomatous masses also present a significant diagnostic burden to most sarcoma centers. Despite efforts to accurately distinguish between benign and malignant entities, including lipomas, atypical lipomatous tumors/well-differentiated liposarcomas, differentiating these entities in the diagnostic pathway and initiating appropriate and individual patient-centered diagnostic and therapeutic strategies remains challenging [4–6]. The recent Delphi-based consensus study of Noebauer-Huhmann et al [7] underscores this challenge and presents a decision tree to minimize the variability in clinical practice in using advanced imaging techniques. Histologically, liposarcomas manifest in four distinct subtypes: atypical lipomatous tumors or, depending on the localization within the body, also known as well-differentiated liposarcoma, myxoid liposarcoma, dedifferentiated and pleomorphic liposarcoma. Each liposarcoma subtype presents a wide range of imaging characteristics and sometimes overlaps with non-neoplastic types [4]. Even histological characteristics, such as fat entrapped within tumors, pose interpretive challenges and could be mistaken for adipocytic differentiation.

Advances in molecular diagnostics and biopsy techniques are promising in refining diagnostic accuracy and reducing the need for unnecessary surgeries [8]. However, hurdles such as sample acquisition, cost and availability considerations, interpretation challenges, and primarily the heterogeneity of lipomatous tumors have made the colloquially termed excision biopsy, also known as “whoops surgery,” not entirely obsolete [9]. This term refers to instances of inadvertent surgical intervention resulting from misinterpretation and could result in increased morbidity and increased local disease recurrence rates [10]. Previous endeavors aimed to establish decision trees but were hindered by limited participation [3].

The present study aimed to evaluate and compare the current standard operating procedures (SOPs) for diagnostic work-up of lipomatous tumors among participating European Organization for Research and Treatment of Cancer (EORTC) sarcoma centers.

## Methods

All members of the Soft Tissue and Bone Sarcoma Group of the EORTC were invited by the chair of the EORTC Sarcoma Group to participate in this study in March 2023 by email. No formal exclusion criteria existed for participation. These experts were asked to provide their institutional guidelines for the management of adipocytic soft-tissue tumors. The following short clinical vignette was provided to all experts as a question: “Which criteria are used at your institution for the decision of surveillance, biopsy, or primary resection of adipocytic soft-tissue tumors? Please describe your institutional standard operating procedures in the otherwise healthy patient.”

Responses were received as text files or diagrams. The responses were transformed into decision trees, including diagnostic criteria, leading to three possible recommendations (follow-up, core needle biopsy, or resection). Some criteria were adjusted to represent uniformity [11]: Criteria mentioned by only one center were not included in the decision tree analysis. Imaging criteria such as calcification, bone density, septation size, and signal intensity were classified as heterogeneous or homogeneous features of imaging. Superficial and deep refer to the location of the mass in relation to the investing fascia. Superficial tumors do not involve the investing fascia. Some centers provided an exact definition of size, some provided a range, and others were descriptive. Size was adjusted as a numeric variable with an arbitrarily determined upper limit of 15 cm during analysis. The decision trees were sent to the experts for validation [12]. All trees were validated by April 2024. The decision trees were subsequently compared using the objective consensus methodology [13]. This method allows evaluation and comparison of individual potential combinations of the diagnostic criteria and recommendations of individual centers.

The level of consent or dissent was then quantified among the individual recommendations and expressed as a percentage. Individual validated decision trees are given in Supplement 1.

Mediastinal and retroperitoneal lipomatous tumors were excluded from the present analysis.

### Statistical analysis

The present data of the decision‐making analysis are presented as consensus percentages among participating centers [13]. Thresholds for low, intermediate, and high consensuses were arbitrarily determined as consensuses of 50%, 75%, or 90% among participating centers. Consensuses smaller than 50% were considered dissent among participating centers.

## Results

### Decision-making criteria for lipomatous tumors

Fourteen members (subsequently referred to as “experts” or “centers”) from their respective centers (Madrid, Spain; Leuven, Belgium; Warsaw, Poland; Cambridge, United Kingdom; Mannheim, Germany; Herzliya, Israel; Düsseldorf, Germany; Göttingen, Germany; Groningen, the Netherlands; Birmingham, United Kingdom; Paris, France; Lausanne, Switzerland; Milan, Italy; and Amsterdam, the Netherlands) agreed to participate. Six diagnostic criteria were analyzed for SOPs in the diagnostic work-up of lipomatous tumors among participating sarcoma centers. These were the presence of heterogeneity features on imaging, superficial tumor location, tumor location in the trunk, tumor size, history of tumor growth, and symptoms related to the tumor. Table 1 shows an overview of the six diagnostic decision criteria used by various centers in their corresponding SOPs. The individual centers were assigned a letter from A to N. The most frequently used decision-making criteria of the various centers of lipomatous tumors were the depth of the lipomatous tumor, imaging features (summarized as heterogeneous features), history of tumor growth, and tumor-related symptoms. Almost all centers (12 out of 14) considered the depth of the lipomatous tumor as a decision-making criterion. Imaging features such as homogeneity, indicating non-aggressive tumors, and heterogeneity, indicating locally aggressive tumors, were also used in the majority (10 out of 14) of centers as diagnostic criteria. Notably, half of the centers did not consider tumor growth in their diagnostic criteria. One center (H) used no criteria but instead recommended biopsy for all lesions for mouse double minute 2 homolog (MDM2) analysis to differentiate atypical lipomatous tumors/well-differentiated liposarcomas from lipoma. Various cut-offs were applied for tumor size. Five centers did not consider size as a diagnostic criterion. Two centers (C and K) defined multiple cut-offs based on whether the lesion was located superficially or deep in the tissue. The remaining nine centers defined cut-offs ranging from 2 to 10 cm to categorize lesion size. Four centers used a cut-off of 3 cm for the size of a lesion. Five centers used the cut-off of 5 cm. Cut-off sizes of 7 and 10 cm were applied by one and two centers, respectively. Generally, larger lesions were more likely to undergo a biopsy.

**Table 1:** Diagnostic decision criteria used by each center

Diagnostic criteria	A	B	C	D	E	F	G	H	I	J	K	L	M	N
Heterogeneity features (y/n)	x	x		x		x	x		x	x	x	x	x	
Superficial (y/n)	x	x	x		x	x	x		x	x	x	x	x	x
Trunk (y/n)											x	x	x	x
Size (s, m, l)	m		s, m		s	m			l		s, l	m	l	s
History of growth (y/n)	x	x	x	x		x	x							x
Symptomatic (y/n)	x	x	x	x					x	x				x

* y* yes, *n* no, *s* small (cut-off of 3 cm), *m* medium (cut-off of 5 cm), *I* large (cut-off of at least 7 cm), x denotes a criterion being used by the respective center

Alphabetical letters from ”A” to ”N” represent the individual anonymized centers in random order

### Decision-making criteria for lipomatous tumors with a high consensus for > 90% of all centers

**Fig. 1:**
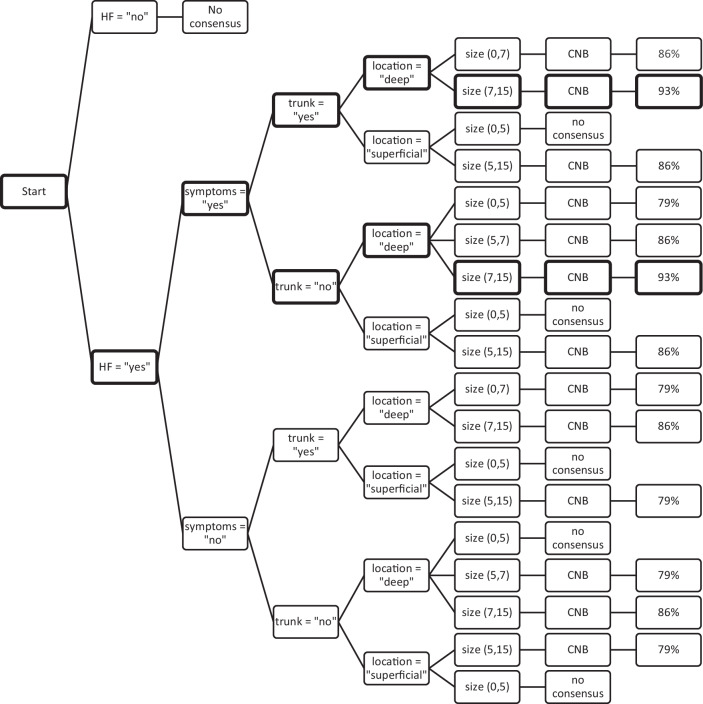
Combination of decision criteria with a consensus rate of at least 75%. An arbitrary lower limit of 0 cm and an upper limit of 15 cm were applied for the size parameter, i.e., [0,5] means lesion size < 5 cm. The combination of decision criteria with a consensus of 93% is highlighted in bold borders. HF, heterogeneity features; CNB, core needle biopsy

The decision tree of most centers comprised multiple decision criteria. Supplement 1 provides an overview of all the validated decision trees. The decision outcomes were either core needle biopsy (CNB), follow-up (FU), or resection. One combination of the various decision-making criteria revealed the highest consensus rate of 93% of all centers. This combination is shown in Fig. 1 and highlighted in bold borders: Lipomatous tumors with a deep location, a tumor size exceeding 7 cm, and associated symptoms were further evaluated with CNB and subsequent pathology assessment as SOP (regardless of the body region location, such as trunk, neck, or extremities).

### Decision-making criteria for lipomatous tumors with an intermediate consensus for > 75% of all centers

Figure 1 shows the outcomes with a consensus of over 75%. There is especially a strong consensus (> 79%) for CNB in heterogeneous symptomatic deep lesions, regardless of size.

The outcomes with a consensus of over 75% usually involved heterogeneous features on imaging that led to the decision to perform a CNB. The exceptions were superficially located heterogeneous lesions with sizes of < 5 cm, regardless of the presence of symptoms, and deep heterogeneous lesions with sizes of < 5 cm without the presence of symptoms. In these cases, no consensus existed.

### Decision-making criteria for lipomatous tumors with an intermediate consensus for > 50% of all centers

Outcomes with a consensus of over 50% usually involved heterogeneous features on imaging and led to the decision to perform a CNB (Supplement 2*)*. The exceptions were superficially located heterogeneous lesions with sizes of < 5 cm, regardless of the presence of symptoms, and deep heterogeneous lesions with sizes of < 5 cm without the presence of symptoms. In these cases, no consensus existed.

### Decision-making criteria for lipomatous tumors with a low consensus of < 50% of all centers

Eleven combinations of decision criteria had no consensus (i.e., fewer than 50% of centers recommended either CNB, FU, or resection). Many decision-making criteria without consensus involved lipomatous tumors without symptoms or those showing homogenous features on imaging. For example, superficial symptomatic lesions with homogenous features on imaging and with no history of growth did not achieve consensus for SOPs among the various centers. One additional combination of decision-making criteria without consensus among centers was lipomatous tumors characterized by homogenous features on imaging, deeply located, not in the trunk, showing neither tumor growth nor symptoms, and a tumor size of 0–5 cm. For this combination of tumor characteristics, CNB, FU, and resection were almost equally recommended; thus, no consensus was noted. An overview of these decision outcomes can be found in Supplement 3.

### Decision-making criteria for lipomatous tumors with a high consensus for FU

**Fig. 2:**
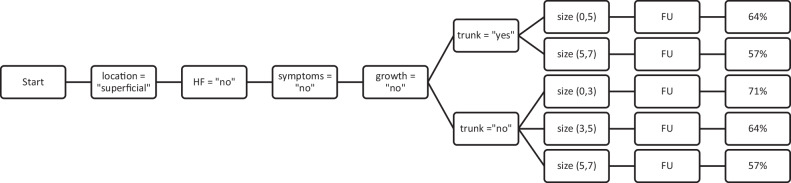
Decision flow where the outcome follow-up reaches a consensus rate of at least 50%. An arbitrary lower limit of 0 cm and an upper limit of 15 cm were applied for the size parameter, i.e., [0,5] means lesion size < 5 cm. HF, heterogeneity features; FU, follow-up

The decision to FU showed the highest consensus (71%) in lipomatous tumors not located in the trunk, smaller than 3 cm, showing neither growth nor symptoms, and exhibiting homogeneous features on imaging. Figure 2 illustrates all the decision combinations with at least 50% consensus for the decision to FU. The SOP leading to a FU is summarized as superficial lesions without growth and homogenous features on imaging. Whether the lesion was in the trunk, neck, or extremities did not affect the SOPs for FU.

### Decision-making criteria for lipomatous tumors with regard to resection

The decision for surgical resection shows low consensus (Supplement 2a). The highest consensus with regards to resection reaches 64% for homogeneous deeply located lesions, not located in the trunk, small size (< 2 cm) and showing growth and symptoms.

## Discussion

This study highlights the decision-making criteria with the highest consensus rate among 14 participating academic sarcoma centers in diagnosing lipomatous tumors: Tumors located deep in the tissue, a tumor size exceeding 7 cm, a tumor with heterogeneous features in imaging, and associated symptoms emerged as pivotal criteria. These were further evaluated with CNB and subsequent pathology assessment as SOP at almost all centers. These criteria still showed very high consensus rates regardless of size. This finding is in accordance with the recent guideline from Noebauer et al [7] where deep-seated tumors, regardless of size, are advised to be referred to a specialized center for CNB.

Five centers did not consider lesion size, and five other centers used a cut-off of 5 cm. A study from Cairncross et al [14] with 248 patients shows no atypical lipomatous tumors smaller than 5 cm. Findings from McClintock et al [15] in 178 patients confirms these findings and showed no malignancy in pathology samples with masses smaller than 5 cm.

By identifying SOPs and integrating expert opinions, the present study may serve as guidance for clinical decision-making in the management of lipomatous tumors, reducing the need for unnecessary interventions.

The decision-making criteria showing a consensus rate of > 75% involved almost always heterogeneous imaging features and led to a CNB. The decision-making criteria showed no consensus involved tumors without symptoms or those with homogenous features on imaging. The decision to FU showed the highest consensus rate in lipomatous tumors that were small, were probably located in the extremities, had neither symptoms nor growth, and showed homogeneous features on imaging. These observations concur with many US-based centers’ findings [15–17].

The decision for primary surgical tumor resection showed overall low consensus. The highest consensus is reached for homogeneous deeply located small lesions outside the trunk, which show growth and symptoms.

The studies by Brisson et al and the recent meta-analysis by Wilson et al [18, 19] both indicate a higher likelihood of finding atypical lipomatous tumors/well-differentiated liposarcomas or dedifferentiated liposarcomas in patients who are older than 60 years, have tumors larger than 10 cm, or have tumors located in the lower limb rather than the upper limb. Additional factors associated with these tumors include the presence of non-fatty areas, thickened septations, enhancing components, and deeper tumor location relative to the fascia.

Interestingly, while Brisson et al identified patient age and tumor location (lower limb vs. upper limb) as important indicators, these were not decision-making criteria for lipomatous tumors at any of the centers surveyed in this study. In contrast, tumor size, heterogeneous features on imaging, and deeper tumor depth were frequently used as decision-making criteria among sarcoma centers. Additionally, tumor-related symptoms were often considered a decision-making criterion by the surveyed sarcoma centers. However, Wilson et al’s meta-analysis did not identify symptoms as a distinguishing factor between atypical lipomatous tumors or malignant liposarcomas and benign lipomatous lesions on CT and MRI.

Noebauer et al published a revised Delphi-derived consensus article involving 46 specialized musculoskeletal radiologists from 12 European countries [7]. The study presents an imaging algorithm for the primary diagnosis of soft tissue mass. The study therefore mainly focuses on which imaging modality to use and provides general criteria for referral to a sarcoma center. These criteria include clinical suspicion and indeterminate imaging as criteria for CNB in a tumor center. Likely benign imaging features are the only criterion for a possible follow-up. According to the findings of Noebauer et al, all indeterminate ultrasound findings are ideally referred to a sarcoma center for further evaluation with MRI. The present study is not entirely comparable with the revised guideline of Noebauer et al, as this study focuses more on diagnostic criteria rather than imaging modality. Besides a difference in aim, there is also a difference in methodology, similar to the study of Moulin et al and explained below in further detail. Moulin et al published a consensus article from eight European centers for a diagnostic strategy for adipocytic soft-tissue tumors in adults [3]. As the decision-making strategy diagram from Moulin et al was obtained by consensus, it was expected that centers participating in that study would refer to this diagram as their SOP. Of the three centers that participated in both studies (Paris, Amsterdam, and Warsaw), only one referred to this diagram as their SOP. The two remaining centers presented a refined SOP where the symptoms and growth of the tumor were also included as diagnostic criteria. One of these two centers did not consider size regarding deeply located tumors, whereas the third center added growth in deeply located tumors as a diagnostic criterion. However, the present study is not entirely comparable to the consensus article on the diagnostic strategy of adipocytic soft-tissue tumors by Moulin et al [3] nor to the revised Delphi-derived consensus article from Noebauer et al [7] due to differences in aims. The present study did not strive to achieve consensus among participants but adopted an observer role, focusing on documenting various perspectives and assessing whether consensus was present. As substantial differences exist in the optimal diagnostic pathway of adipocytic soft-tissue tumors, this approach appeared suitable for obtaining an understanding of the diverse viewpoints. This contrasts with the Delphi-based consensus striving methodology [20–22], where multiple rounds of anonymous data collection and structured panelist feedback were iterated toward convergence of opinions among carefully selected participants [20–23]. The Delphi-based methodology actively strives to achieve consensus [20–23]. The variations in the optimal SOP are characterized by the complex differentiation among various subtypes of soft-tissue tumors: Approximately 35% of sarcomas are liposarcomas, whereas atypical lipomatous tumors and well-differentiated liposarcomas are the most frequent histological subtype, representing almost half of all liposarcomas [5]. Interestingly, neither conventional histopathology nor imaging provides reliable differentiation between lipomas and their locally aggressive variants. Amplification of MDM2, a crucial molecular marker, is key to distinguishing benign lipomas and their variants from locally aggressive counterparts such as atypical lipomatous tumors and well-differentiated liposarcomas [5]. However, this practice is not universally accepted for small masses (< 5 cm) without suspicious characteristics [15] and has limitations such as availability and sample quality. It is worth mentioning a diagnostic accuracy of 81% for CNB for tumors diagnosed as atypical or well-differentiated lipomatous tumors, owing largely to the heterogeneity of often large lesions [24].

Efforts to improve diagnostic accuracy in the differentiation of malignant lipomatous tumors from lipomas using radiomics features on CT and MRI are a noteworthy development. A radiomics model from a Dutch center [25] based on T1- and T2-weighted MRI achieved an area under the curve (AUC) of 0.89, sensitivity of 0.74, and specificity of 0.88, outperforming three expert radiologists. A more recent study from Peking evaluated a radiomics nomogram based on contrast-enhanced CT, which showed an AUC of 0.861 in external validation [26].

Both atypical lipomatous tumors and well-differentiated liposarcomas exhibit similarities in gross pathology, histopathology, and molecular pathology, presenting as locally aggressive neoplasms. They are referred to as atypical lipomatous tumors in the extremities and as well-differentiated liposarcomas in the mediastinum and retroperitoneum [5]. This location-dependent terminology is associated with differences in long-term prognosis; however, the SOPs for well-differentiated liposarcomas were beyond the scope of this study.

Notably, significant discrepancies in SOPs existed not only between European centers but also between centers in the same country, suggesting the influence of localized preferences [27, 28]. These findings underscore the complexity of standardization efforts in diagnosing lipomatous tumors.

Similar challenges in differentiating enchondromas, atypical cartilaginous tumors, and low-grade chondrosarcomas on imaging and histopathology are well-known. Unsurprisingly, five radiologists from four continents provided various management recommendations for an enchondroma with a tumor size of 5.1 cm in the distal femur [1, 29]. The management recommendations for the clinical vignette of the 5.1 cm enchondroma varied between no management recommendation to management recommendations such as clinical FU, imaging FU on radiographs and MRI, referral to an orthopedic oncologist at a tertiary care hospital, and even tumor curettage of the enchondroma [29].

Our study has limitations. Fourteen European experts from academic centers participated. These 14 experts were active in large academic teaching hospitals and may reflect only a fraction of these European centers. A bias may have existed against the perspectives of experts outside and inside Europe, as well as those working in a non-academic setting, limiting the generalizability of the findings to a broader international context [22, 23]. Additionally, the adaptation of decision trees to represent SOPs requires simplification, potentially overlooking nuances in clinical decision-making. For example, the decision for FU or resection also depended on patient choice in some centers. In these cases, and for algorithmic purposes, the decision trees were simplified according to the purpose of the study to analyze diagnostic workups for lipomatous tumors. Another noteworthy limitation is that imaging modality was not differentiated in the assessment of lesion heterogeneity. One may speculate that criteria for heterogeneity on MRI are assessed differently than on ultrasound or on CT images.

In conclusion, the present study found that the highest consensus for biopsy was for deep large tumors with associated symptoms. For follow-up, consensus was shown for small homogenous tumors not located in the trunk, without growth or symptoms. Consensus on resection, albeit low, involved homogeneous deeply located small tumors outside the trunk with growth and symptoms.

## Supplementary information

ELECTRONIC SUPPLEMENTARY MATERIAL

## Data Availability

Raw data necessary for replication of study findings can be found in the supplementary materials attached as a PDF.

## Abbreviations

AUC: Area under the curve
CNB: Core needle biopsy
EORTC: European Organization for Research and Treatment of Cancer
FU: Follow-up
HF: Heterogeneity features
l: Large
m: Medium
MDM2: Mouse double minute 2 homolog
n: No
s: Small
SOP: Standard operating procedure
y: Yes
