# Supplementary material for: Diagnostic work-up of lipomatous tumors: a decision-making analysis among European sarcoma centers
Source: Insights Imaging. 2025 Jun 14;16:123. doi: 10.1186/s13244-025-02012-7 (PMC12167213; doi:10.1186/s13244-025-02012-7)
Supplement: Supplementary file 1 — ELECTRONIC SUPPLEMENTARY MATERIAL [file 13244_2025_2012_MOESM1_ESM.pdf]

Diagnostic work-up of lipomatous tumors: A decision-making analysis among European sarcoma centers

ELECTRONIC SUPPLEMENTARY MATERIAL

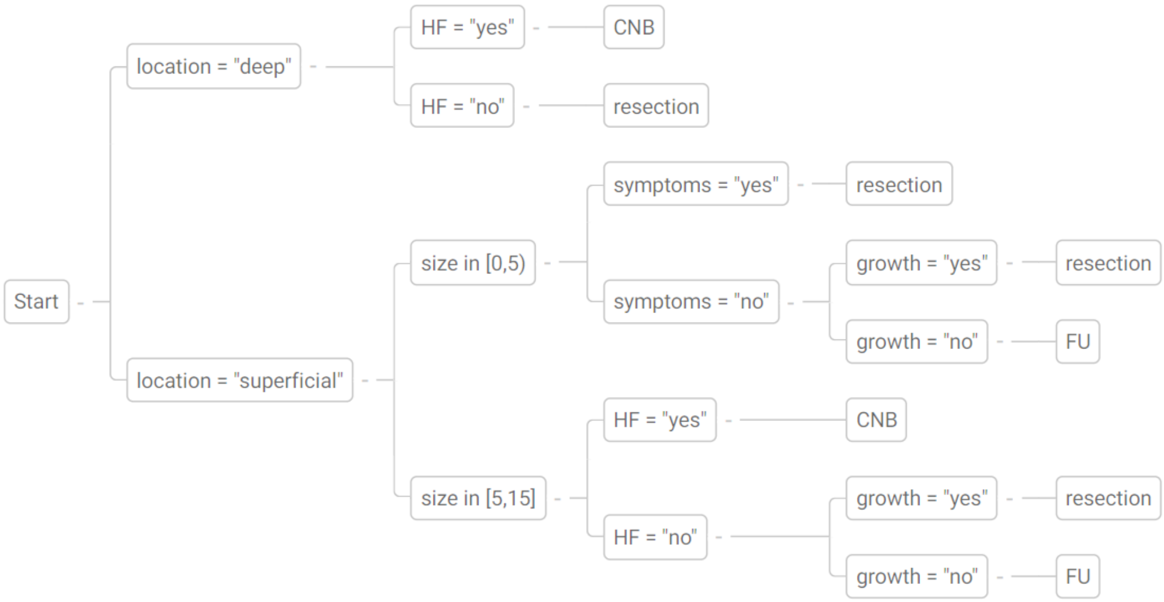

Supplement 1A: A validated decision tree of center A (Amsterdam). An arbitrary lower limit of 0 centimeter and an upper limit of 15 centimeter was applied for the size parameter, i.e. [0,5] means size < 5 cm.

Abbreviations: HF=heterogeneity features, CNB = core needle biopsy, FU = follow-up.

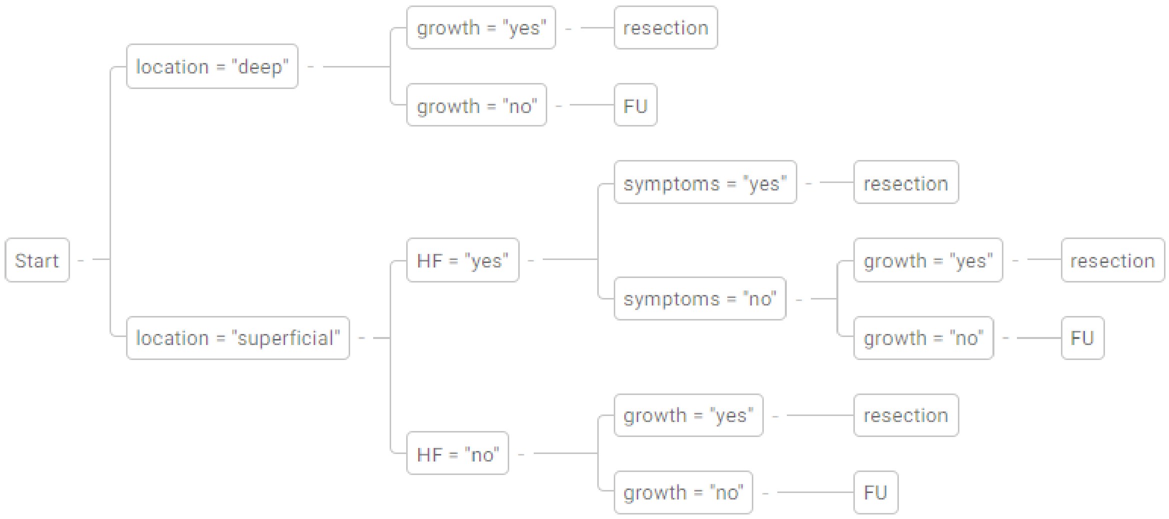

Supplement 1B: A validated decision tree of center B (Düsseldorf).

Abbreviations: HF=heterogeneity features, FU = follow-up.

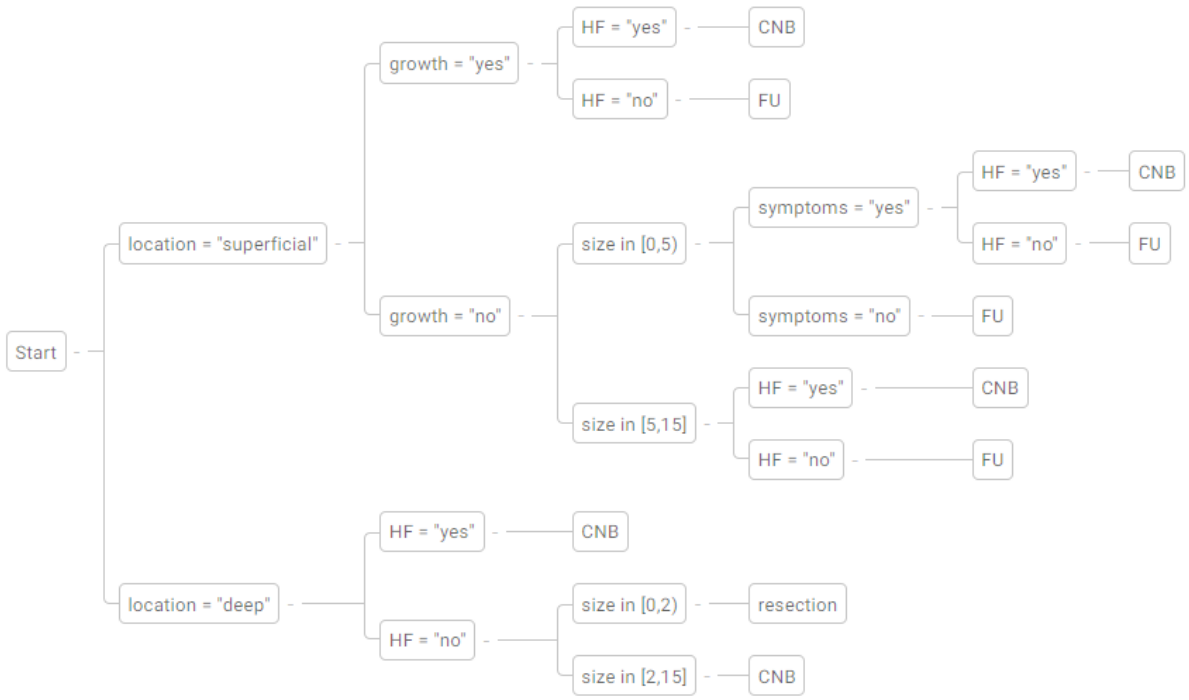

Supplement 1C: A validated decision tree of center C (Cambridge). An arbitrary lower limit of 0 centimeter and an upper limit of 15 centimeter was applied for the size parameter, i.e. [0,5] means size < 5 cm.

Abbreviations: HF=heterogeneity features, CNB = core needle biopsy, FU = follow-up.

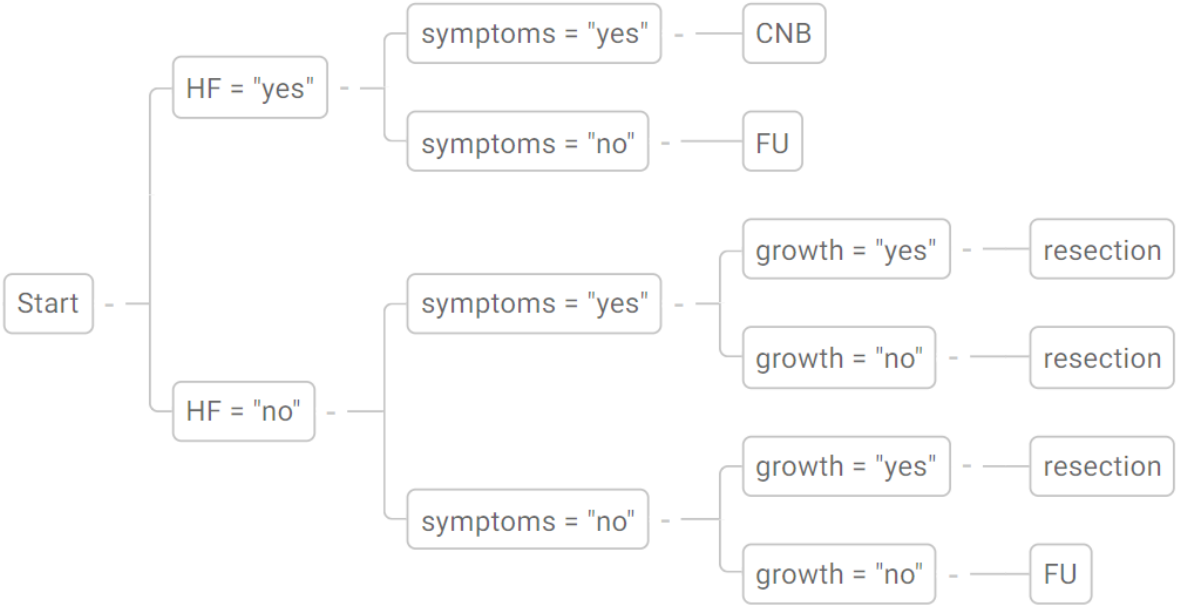

Supplement 1D: A validated decision tree of center D (Groningen).

Abbreviations: HF=heterogeneity features, CNB = core needle biopsy, FU = follow-up.

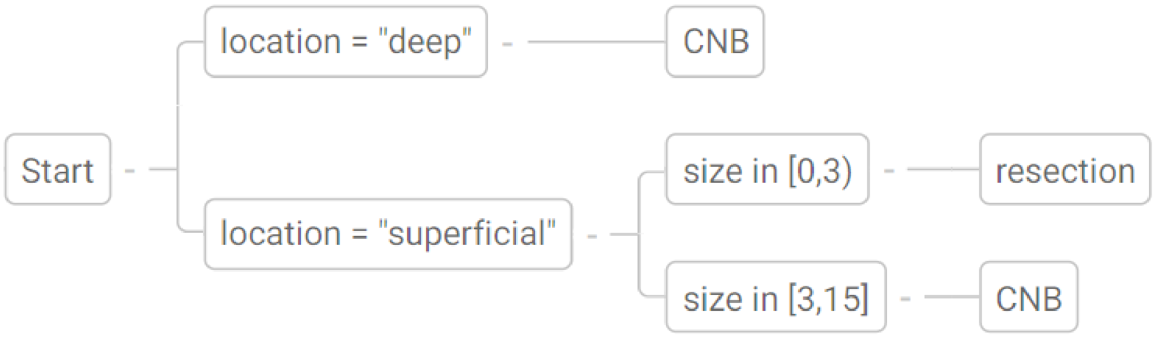

Supplement 1E: A validated decision tree of center E (Göttingen). An arbitrary lower limit of 0 centimeter and an upper limit of 15 centimeter was applied for the size parameter, i.e. [0,3] means size < 3 cm.

Abbreviations: HF=heterogeneity features, CNB = core needle biopsy

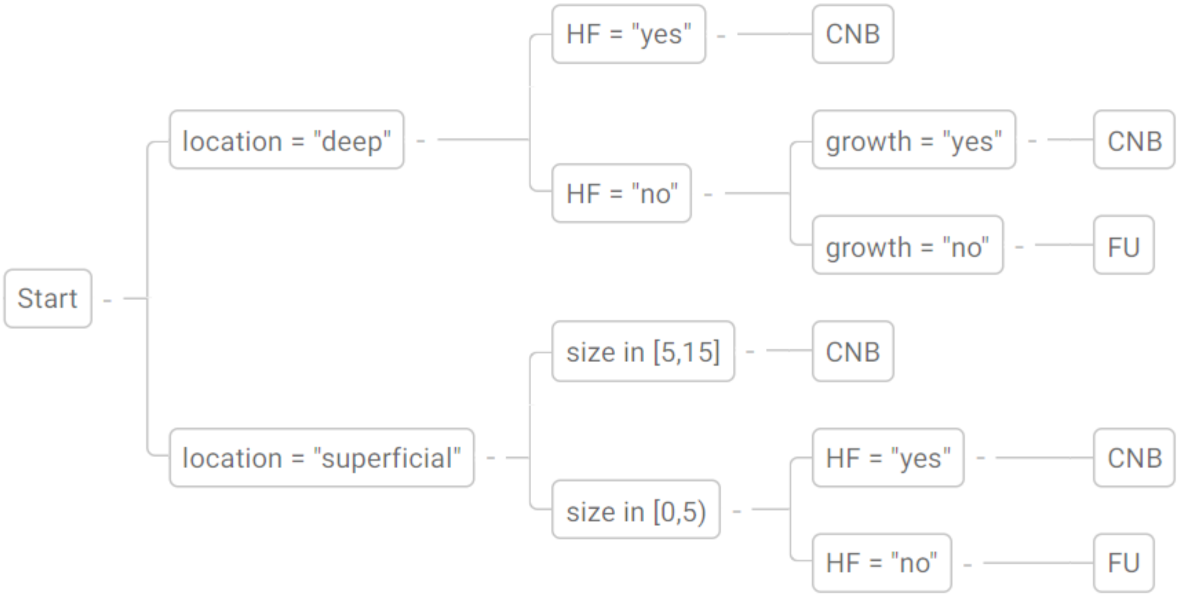

Supplement 1F: A validated decision tree of center F (Warsaw). An arbitrary lower limit of 0 centimeter and an upper limit of 15 centimeter was applied for the size parameter, i.e. [0,5] means size < 5 cm.

Abbreviations: HF=heterogeneity features, CNB = core needle biopsy, FU = follow-up.

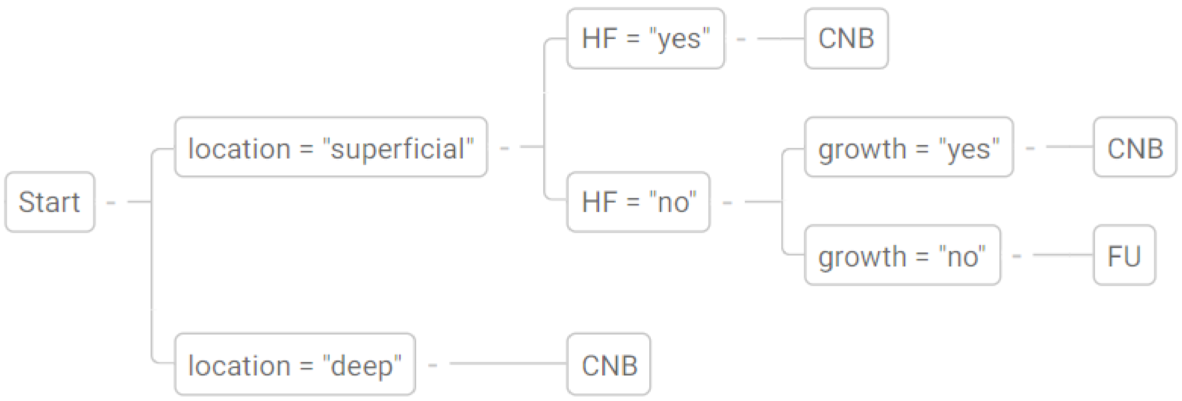

Supplement 1G: A validated decision tree of center G (Lausanne).

Abbreviations: HF=heterogeneity features, CNB = core needle biopsy, FU = follow-up.

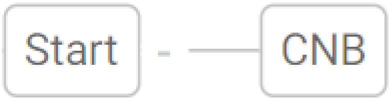

Supplement 1H: A validated decision tree of center H (Madrid).

Abbreviations: CNB = core needle biopsy

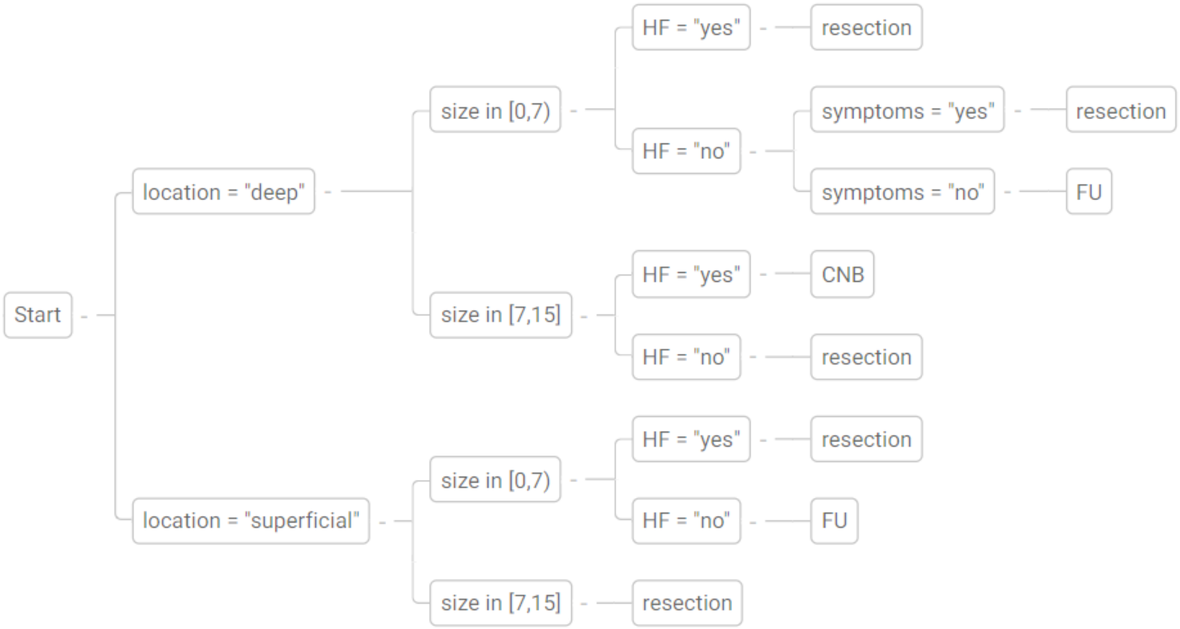

Supplement 1I: A validated decision tree of center I (Birmingham). An arbitrary lower limit of 0 centimeter and an upper limit of 15 centimeter was applied for the size parameter, i.e. [0,7] means size < 7 cm.

Abbreviations: HF=heterogeneity features, CNB = core needle biopsy, FU = follow-up.

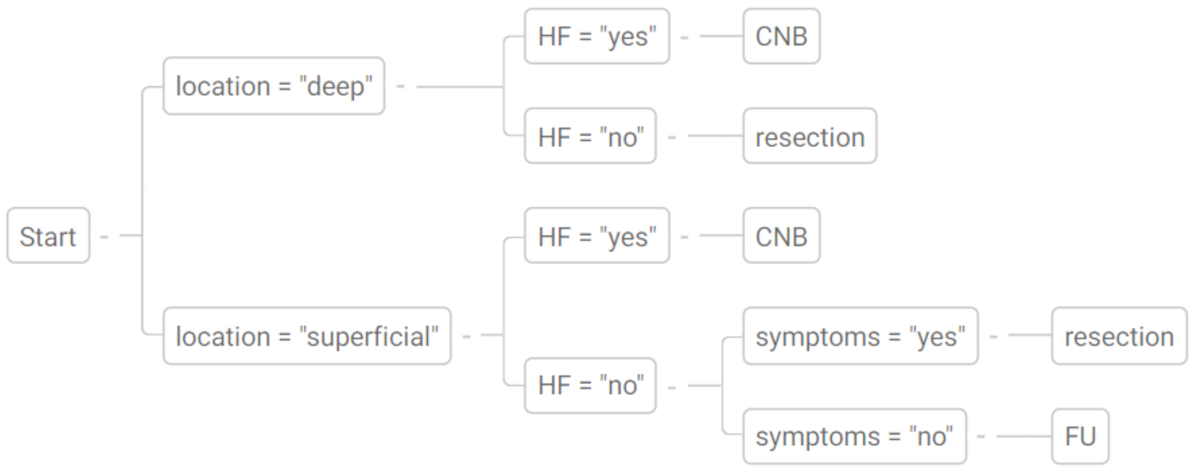

Supplement 1J: Example of a validated decision tree of center J (Milan).

Abbreviations: HF=heterogeneity features, CNB = core needle biopsy, FU = follow-up.

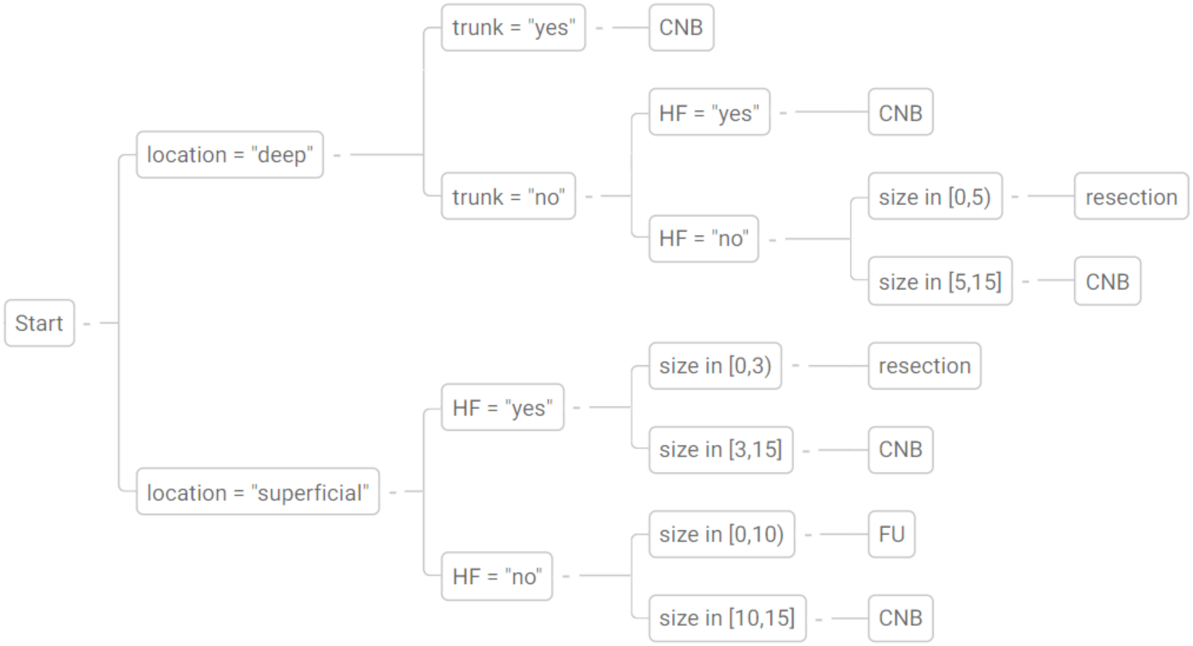

Supplement 1K: A validated decision tree of center K (Paris). An arbitrary lower limit of 0 centimeter and an upper limit of 15 centimeter was applied for the size parameter, i.e. [0,3] means size < 3 cm.

Abbreviations: HF=heterogeneity features, CNB = core needle biopsy, FU = follow-up.

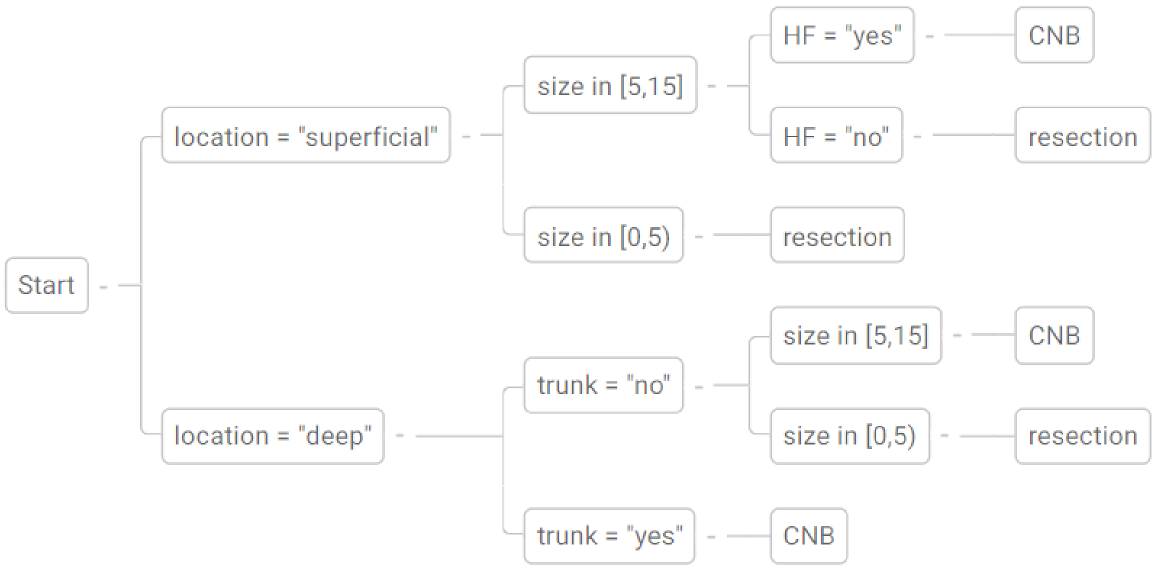

Supplement 1L: A validated decision tree of center L (Leuven). An arbitrary lower limit of 0 centimeter and an upper limit of 15 centimeter was applied for the size parameter, i.e. [0,5] means size < 5 cm.

Abbreviations: HF=heterogeneity features, CNB = core needle biopsy, FU = follow-up.

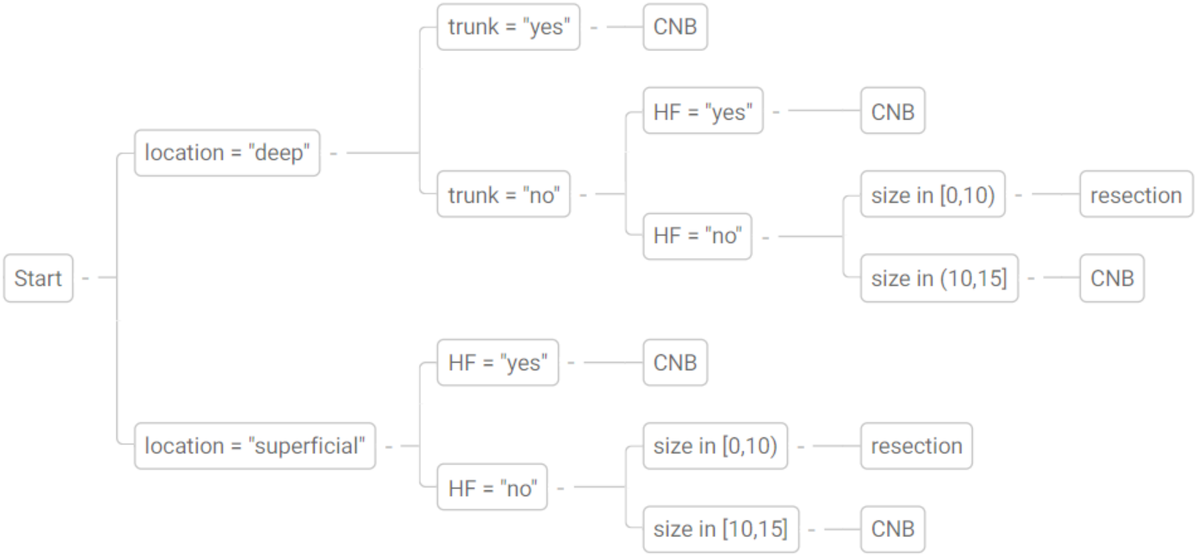

Supplement 1M: Example of a validated decision tree of center M (Mannheim). An arbitrary lower limit of 0 centimeter and an upper limit of 15 centimeter was applied for the size parameter, i.e. [0,10] means size < 10 cm.

Abbreviations: HF=heterogeneity features, CNB = core needle biopsy

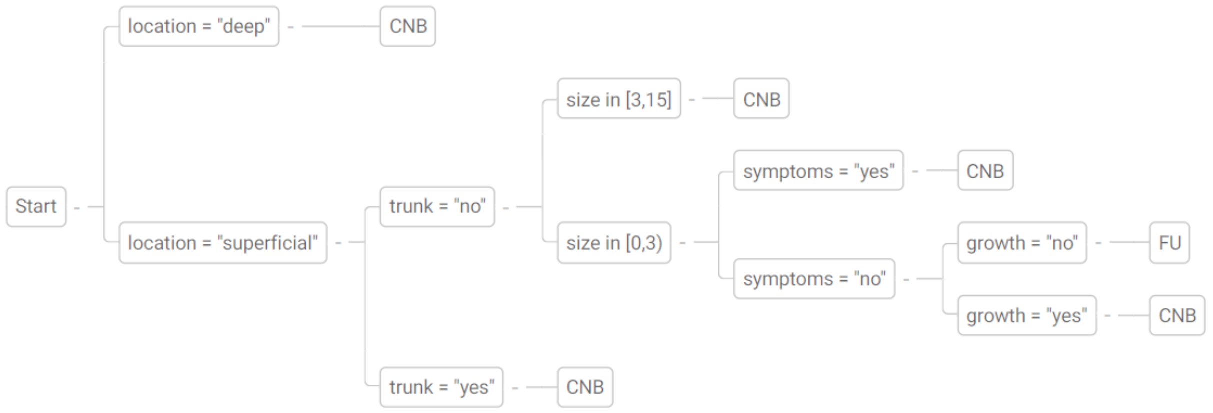

Supplement 1N: A validated decision tree of center N (Herzliya). An arbitrary lower limit of 0 centimeter and an upper limit of 15 centimeter was applied for the size parameter, i.e. [0,3] means size < 3 cm.

Abbreviations: HF=heterogeneity features, CNB = core needle biopsy, FU = follow-up.

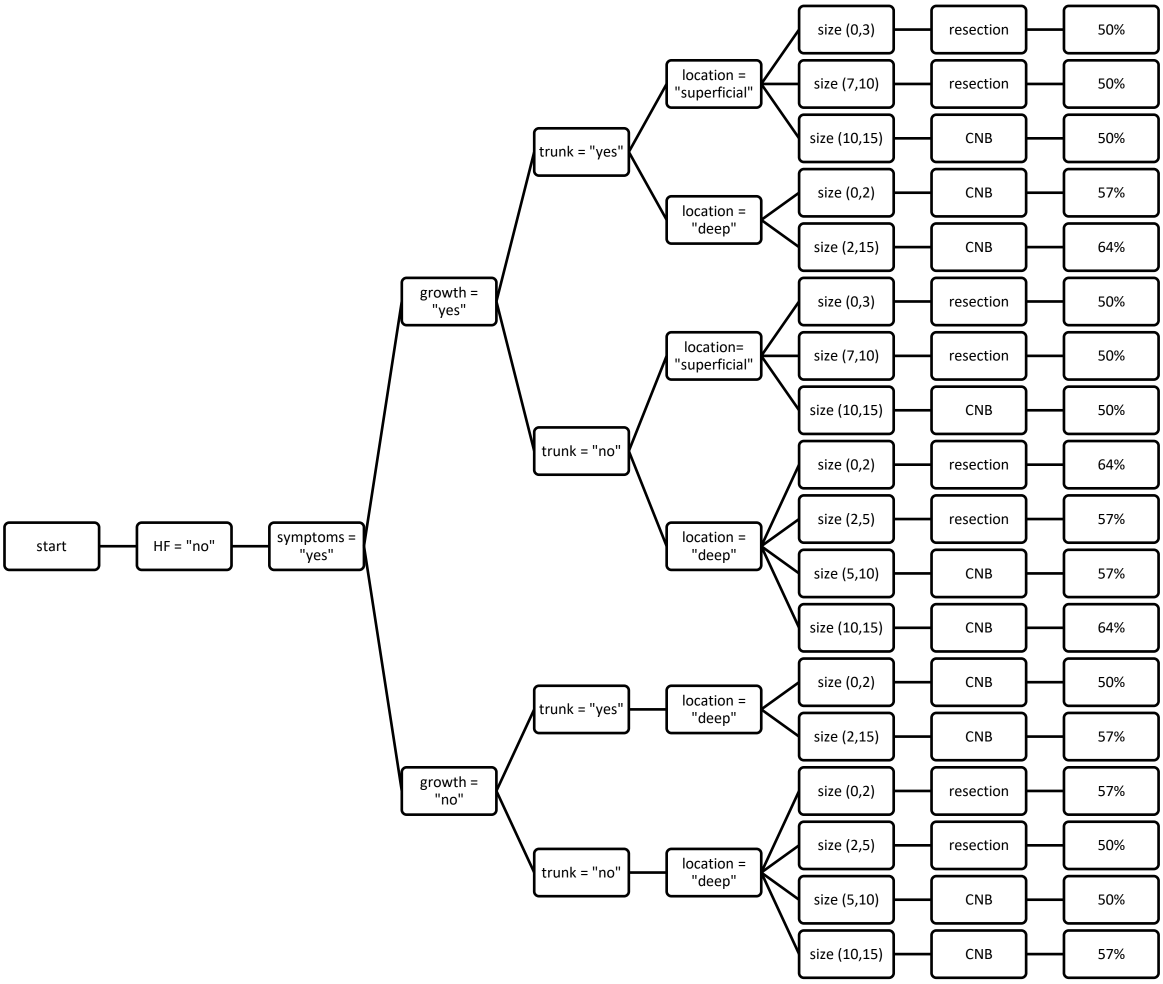

Supplement 2A: Combination of decision criteria with a consensus rate between 50-75%.

An arbitrary lower limit of 0 cm and an upper limit of 15 cm were applied for the size parameter, i.e., [0,5] means lesion size <5 cm.

Abbreviations: HF = heterogeneity features, CNB = core needle biopsy, FU = follow-up.

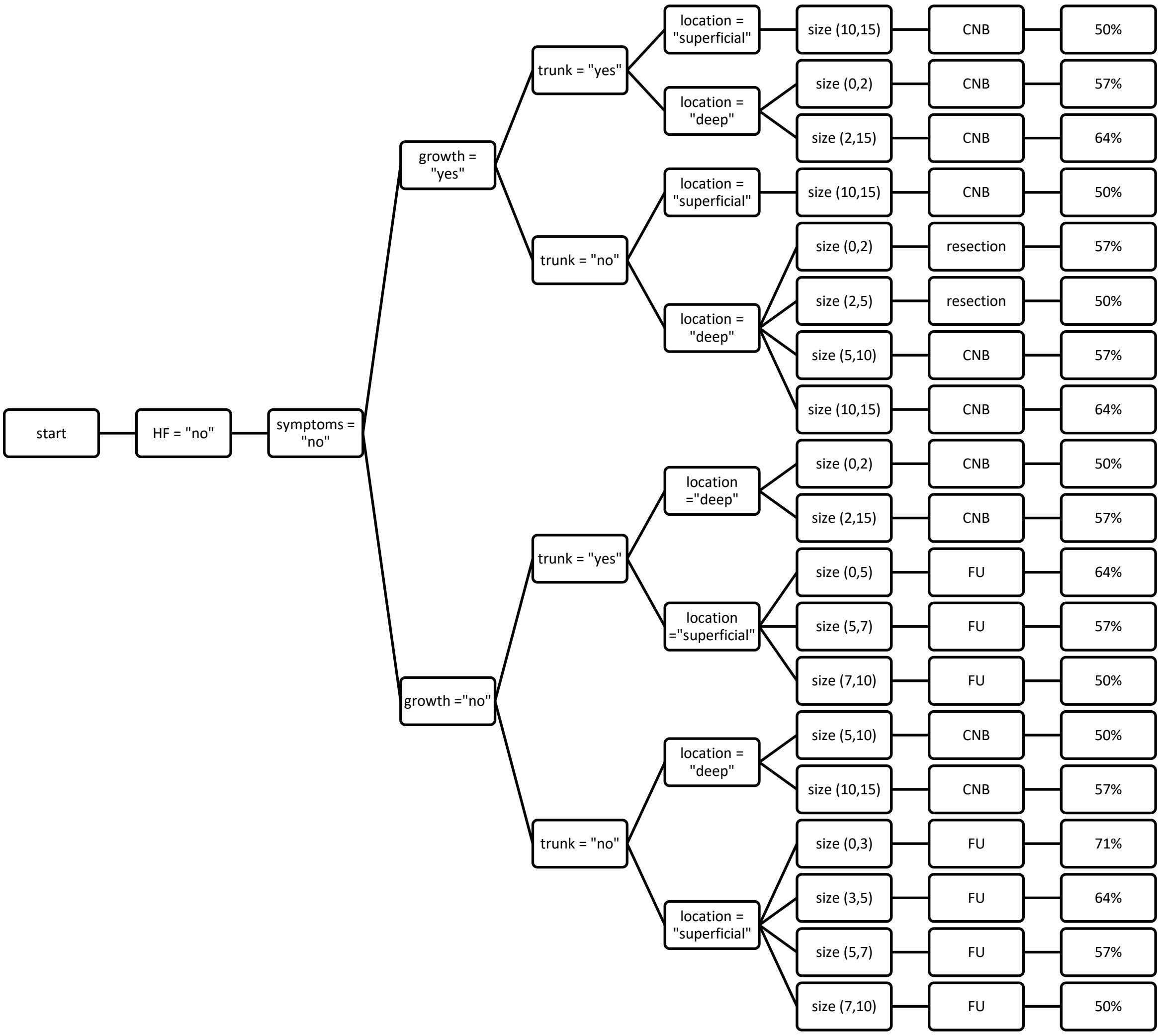

Supplement 2B: Combination of decision criteria with a consensus rate between 50-75%.

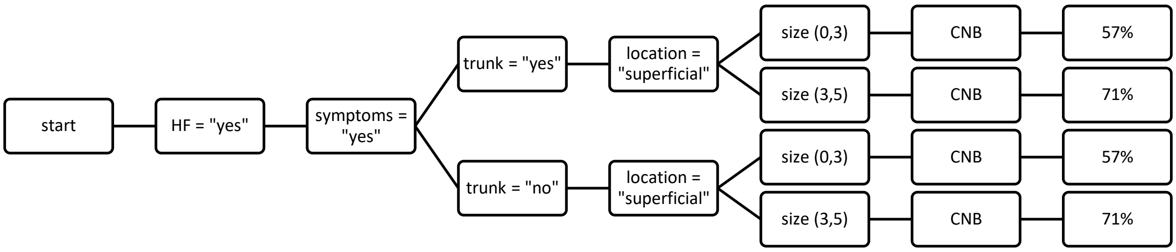

Supplement 2C: Combination of decision criteria with a consensus rate between 50-75% (continued).

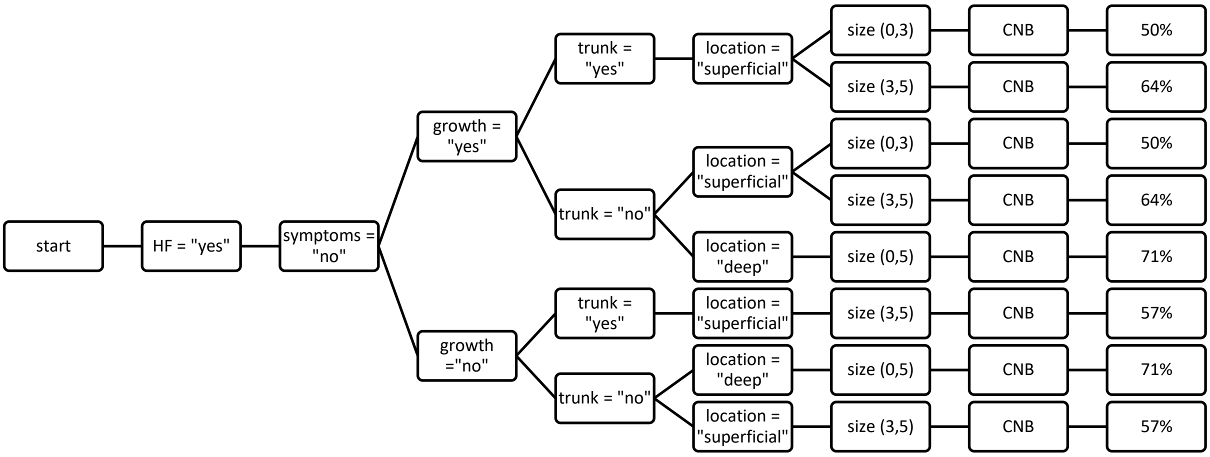

Supplement 2D: Combination of decision criteria with a consensus rate between 50-75% (continued).

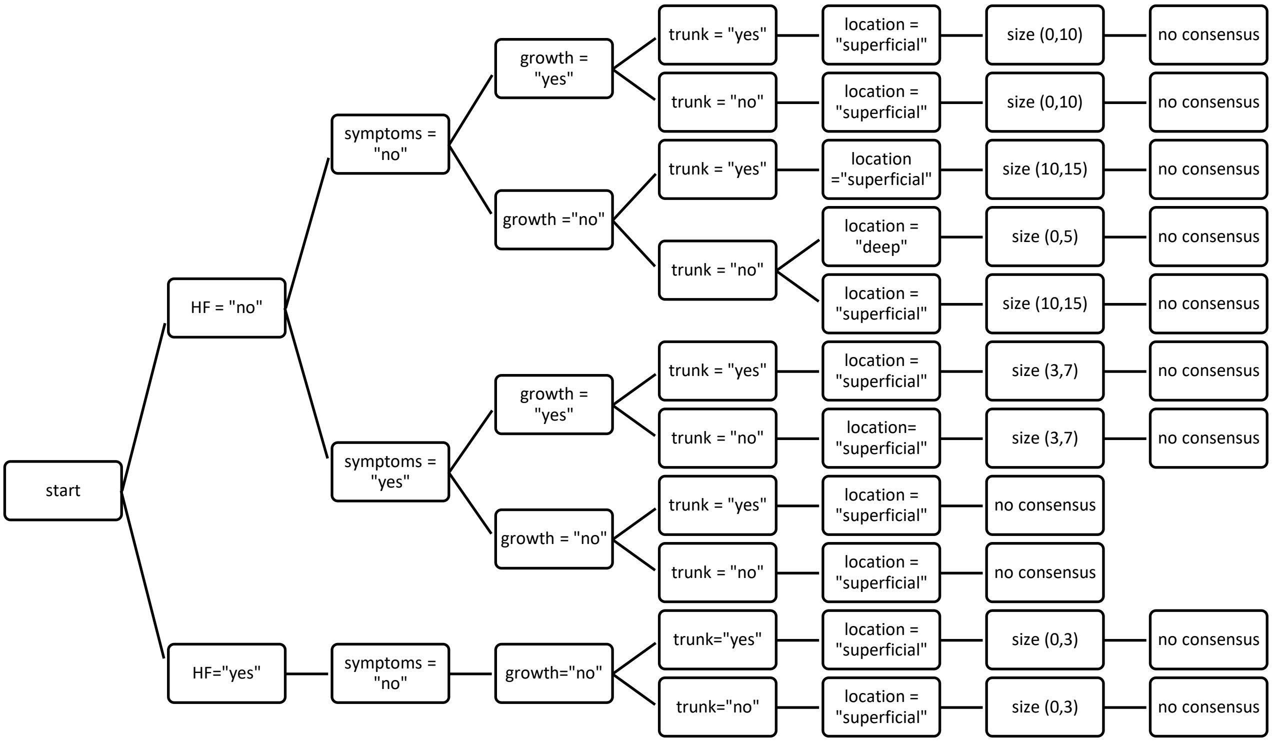

Supplement 3: Combination of decision criteria with a consensus rate lower than 50%, i.e. no consensus.
